# Discovery of long non-coding RNAs in the liver fluke, *Fasciola hepatica*

**DOI:** 10.1371/journal.pntd.0011663

**Published:** 2023-09-28

**Authors:** Paul McVeigh, Erin McCammick, Emily Robb, Peter Brophy, Russell M. Morphew, Nikki J. Marks, Aaron G. Maule

**Affiliations:** 1 School of Biological Sciences, Queen’s University Belfast, Northern Ireland, United Kingdom; 2 Department of Life Sciences, Aberystwyth University, Wales, United Kingdom; Consejo Nacional de Investigaciones Cientificas y Tecnicas, Fundación Mundo Sano, ARGENTINA

## Abstract

Long non-coding (lnc)RNAs are a class of eukaryotic RNA that do not code for protein and are linked with transcriptional regulation, amongst a myriad of other functions. Using a custom *in silico* pipeline we have identified 6,436 putative lncRNA transcripts in the liver fluke parasite, *Fasciola hepatica*, none of which are conserved with those previously described from *Schistosoma mansoni*. *F*. *hepatica* lncRNAs were distinct from *F*. *hepatica* mRNAs in transcript length, coding probability, exon/intron composition, expression patterns, and genome distribution. RNA-Seq and digital droplet PCR measurements demonstrated developmentally regulated expression of lncRNAs between intra-mammalian life stages; a similar proportion of lncRNAs (14.2%) and mRNAs (12.8%) were differentially expressed (p<0.001), supporting a functional role for lncRNAs in *F*. *hepatica* life stages. While most lncRNAs (81%) were intergenic, we identified some that overlapped protein coding loci in antisense (13%) or intronic (6%) configurations. We found no unequivocal evidence for correlated developmental expression within positionally correlated lncRNA:mRNA pairs, but global co-expression analysis identified five lncRNA that were inversely co-regulated with 89 mRNAs, including a large number of functionally essential proteases. The presence of micro (mi)RNA binding sites in 3135 lncRNAs indicates the potential for miRNA-based post-transcriptional regulation of lncRNA, and/or their function as competing endogenous (ce)RNAs. The same annotation pipeline identified 24,141 putative lncRNAs in *F*. *gigantica*. This first description of lncRNAs in *F*. *hepatica* provides an avenue to future functional and comparative genomics studies that will provide a new perspective on a poorly understood aspect of parasite biology.

## Author summary

With increased understanding of genomes and transcriptomes it has become clear that not all RNAs code for protein, in contrast to the Central Dogma of molecular biology. Nevertheless, many such “non-coding” RNAs have functions in their own right. For example, long non-coding (lnc)RNAs comprise a large proportion of the human genome and control expression of some human protein coding genes. In parasites, lncRNAs remain understudied and poorly understood. This paper provides the first description of the lncRNA complement of the liver fluke, *Fasciola hepatica*. We show that lncRNA expression changes as parasites develop, in a similar manner to protein-coding mRNA, suggesting important roles in parasite growth and development. Some lncRNAs show correlated developmental expression with mRNAs coding for important parasite proteins, suggesting that they may interact, suggesting regulatory functions for fluke lncRNAs. A larger subset of lncRNA may interact with micro (mi)RNAs, another type of non-coding RNA that are important regulators of gene expression in eukaryotes. These interactions are, again, important additions to our understanding of fluke lncRNA biology. Our ultimate goal is to discover ways to inhibit these interactions that could be used for new medicines for fluke infections which are a global scourge on the farming industry.

## Introduction

*Fasciola* spp. liver fluke are helminth parasites that impact the health and productivity of farm animals, leading to considerable costs for international agricultural economies [1–3]. *Fasciola* also infects humans and is recognized as a zoonotic Neglected Tropical Disease (NTD) pathogen [4]. Fluke pathogenicity is compounded by the prevalence of fluke field populations resistant to key anthelmintics including albendazole, clorsulon, oxyclozanide, rafoxanide and triclabendazole [5,6] and increased prevalence driven by predictions of warmer, wetter, weather patterns associated with the climate crisis [7].

Efforts to develop new treatments for liver fluke infections have been supported by the development of systems-level experimental resources over the past decade, including genome, transcriptome, proteome and functional genomics methodologies for *F*. *hepatica* [8]. This increased volume and resolution of ‘omics data has yielded insights into the non-coding (nc)RNAs of *Fasciola* genomes, where (mi)RNA complements are beginning to be clarified [9–20]., including a subset that appears to be secreted within extracellular vesicles (EVs). Transfer RNA fragments are the only other ncRNA to have been identified from *F*. *hepatica* transcriptomes [17], but eukaryotic transcriptomes also contain additional ncRNA families that remain poorly characterised. One of the most widely studied over recent years has been the long non-coding (lnc)RNAs, arbitrarily defined as transcripts longer than 200nt that do not code for protein. Interest in this family has largely been driven by perceived importance in human tumour biology [21–23], however increasing numbers of studies are beginning to describe these molecules in non-human animal systems, as well as plants, yeast and prokaryotes [24–29]. The effects of lncRNAs on expression of protein coding transcripts is well recognised, where lncRNAs can regulate expression of overlapping or adjacent protein coding transcripts [30,31].

Amongst lncRNA functions are roles in transcription, translation, protein localization, cellular integrity, the cell cycle, apoptosis, and stem cell pluripotency (for review see [32]). These functions were identified by RNA interference (RNAi) experiments, focusing on cytosolic lncRNAs in human systems [33,34], and increasingly, CRISPR-based perturbation of lncRNA expression in human cell lines [35], and mice [36]. This volume of functional data means that mammalian experimental systems remain our touchstone for understanding lncRNA biology. Functional genomics methods have been less widely applied to invertebrates, but RNAi has yielded functional data in arthropods [37,38] while CRISPR has yielded lncRNA functional insights in *C*. *elegans* [39,40]. The first lncRNA sequence datasets have now been published from parasitic flatworms, including *Schistosoma mansoni* and *S*. *japonicum* [41–45], *Echinococcus granulosus* [46] and *Macrostomum lignano* [47], and now include the first reports of lncRNA functions in these species [48]. LncRNAs are now being recognised as potential therapeutic targets for new anti-parasitic drugs [49], and rapid progress is being made in development of lncRNA-targeting, oligonucleotide-based drugs for several human diseases [50,51].

LncRNAs are appealing drug targets because they tend to have tightly regulated expression in space and time, and appear to show low conservation between parasite and host [49,52]. These characteristics could imply a lower probability of off-target effects than protein-targeting drugs. LncRNAs could also be targeted by oligonucleotide-based drugs, which have a much lower development cost than small-molecule based therapies [53]. However, this appeal is mitigated somewhat by difficulties in formulating and administering oligonucleotide drugs to specific locations in the target organism–most are not efficiently absorbed by the intestine so must be delivered parenterally [52].

This paper aimed to provide the first description of long non-coding (lnc)RNAs in *F*. *hepatica*, and generate insights into fluke lncRNA biology. We achieved this aim, showing that putative lncRNAs are: (i) Developmentally regulated during intra-mammalian development of *F*. *hepatica*; (ii) Expressed in correlation with mRNAs, including proteases important for fluke survival, and (iii) Contain binding sites for miRNAs, suggesting that these two non-coding RNA families may have the capacity to interact in liver fluke. These data provide important insights into potential functions for lncRNAs and will form the basis for future functional genomics studies. Our hope is that these data will catalyse new avenues towards studying, and new perspectives on, liver fluke biology.

## Methods

### Transcriptome assemblies

**Fig 1:**
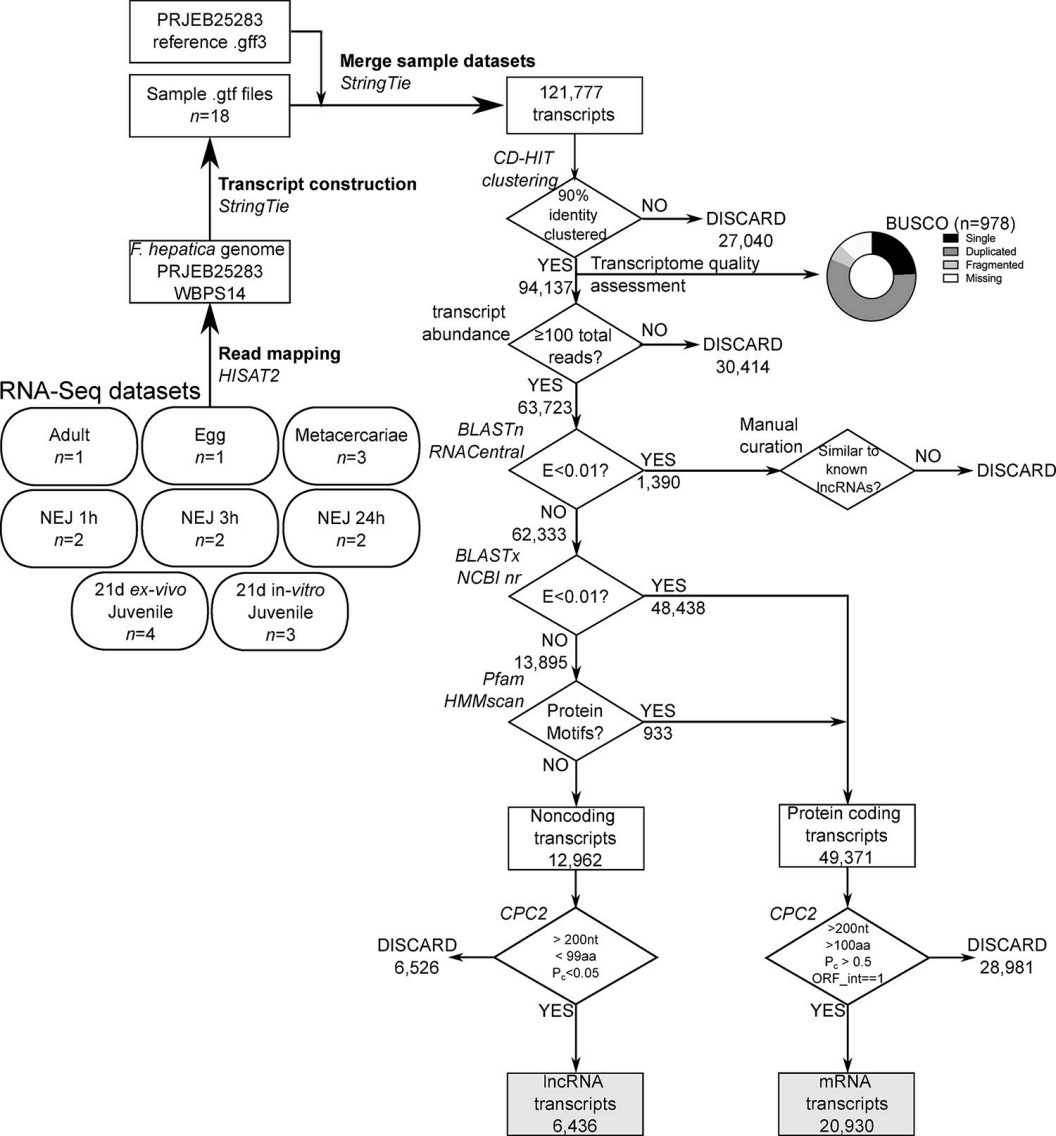
Long non-coding RNA discovery pipeline. Flowchart indicates processing and analysis of RNA-Seq Illumina short read datasets into lncRNA data. Numbers of sequences passing or failing each filter stage of the pipeline are indicated, software tools are named in italics.

Workflow was as described in Fig 1. We began by assembling a non-redundant transcriptome from available *F*. *hepatica* RNA-Seq datasets (18 biological replicate libraries). These included transcriptome data first published by Cwiklinski et al. [54], consisting of non-stranded, paired-end Illumina sequencing read sets from *F*. *hepatica* metacercariae (met; *n =* 3), newly excysted juveniles (NEJ) (1h (nej1h; *n =* 2), 3h (nej3h; *n =* 2), 24h (nej24h; *n =* 2) *in vitro*), *in vivo* liver stage parasites recovered from rats at 21 days post oral exposure (juv21d; *n =* 1), adult parasites (Ad; *n =* 1), and eggs (egg; *n =* 1). These fastq files were obtained from the European Nucleotide Archive (ENA) via project PRJEB6904. We supplemented these with stranded paired-end Illumina sequencing datasets for rat-derived juv21d worms (juv21d; *n = 3*), and 21 day *in vitro* maintained worms (invitro21d; *n* = 3). These six libraries are available from ENA project PRJEB49655, samples ERS9656160-ERS9656165, generated as described [19]. The ftp addresses for all libraries used are provided in S1 Dataset. *F*. *gigantica* lncRNA identification used sequencing datasets from NCBI BioProject PRJNA350370 (S1 Dataset) [55] and an 18h NEJ RNA-Seq dataset [56]. The latter is available from DDBJ/EMBL/GenBank accession GJHP01000000 and can be interrogated *via* https://sequenceserver.ibers.aber.ac.uk/.

Fastq files were mapped against the *F*. *hepatica* genome (WormBase ParaSite PRJEB25283, database version WBPS14) using HISAT2, with a merged transcript assembly generated through StringTie ([57]; using default parameters throughout). This produced 121,277 transcripts of ≧200 nt length (the default length cutoff within StringTie), which were passed into our lncRNA annotation pipeline (Fig 1). A separate stranded transcriptome was also produced by mapping the three [19] juv21d datasets against the *F*. *hepatica* genome as described above, and employing the—*fr* option in StringTie to denote a stranded library in “fr-firststrand” format, matching the dUTP-based synthesis method of the stranded library synthesis kit that we used [19].

### Identification of *F*. *hepatica* lncRNAs

After removal of duplicate transcripts using CD-HIT (using the *cd-hit-est* program, with sequence identity threshold set to 0.9; [58]) (Fig 1), and application of an expression cutoff of ≥100 reads per transcript, contaminating non-coding sequences (ribosomal and transfer RNAs) were removed by comparison with the RNAcentral dataset [59]. Forty sequences possessing similarity (e value < 0.05) with known lncRNAs were replaced back into the dataset. The remaining 63,805 sequences were filtered for similarity with proteins using BLASTx, performed locally using DIAMOND BLAST [60] against the NCBI nr protein sequence database. Transcripts scoring E<0.01 (44,713 sequences) were filtered into a ‘coding sequence’ bin. Remaining sequences (19,092) were screened for protein motifs using Pfam hmmscan, performed locally, resulting in removal of an additional 455 transcripts with motif hits above the default inclusion threshold. The remaining 18,637 presumed non-coding sequences were all analysed with Coding Potential Calculator (CPC2 [61]) in a process of manual cross-checking. This final step ensured that our lncRNAs all had ‘lncRNA-like’ characteristics (>200nt length, encoding ≤100 aa peptide, defined by CPC2 as “noncoding”, with a coding probability of <0.05). Our final dataset contained 7,497 lncRNA transcripts from 6,321 genomic loci. Fig 1 also describes a parallel pipeline for identification of coding RNAs (presumed “mRNAs”), all of which were finally assessed by CPC2 as ‘coding’, with coding probability ≥0.95, with transcript length ≥200nt, and encoded peptides ≥100 aa), yielding 21,697 mRNA transcripts from 9,046 loci. GO analysis was performed for coding RNAs by searching gene IDs within the gene ontology resource (http://geneontology.org/).

### Differential expression analyses

To identify transcripts with statistically significant differential expression (DE) between sequential life cycle stages, we used pairwise exact tests within the EdgeR package [62,63]. Only life stages supported by at least 2 biological replicates were used for these tests (pairwise tests performed: met vs nej1h; nej1h vs nej3h; nej3h vs nej24h; nej24h vs juv21d; juv21d vs met). Raw count data, recovered from StringTie using the prepDE.py script described in the StringTie manual, were piped into EdgeR for analysis. EdgeR output files were parsed using custom Python scripts for list comprehension, allowing extraction of fold change data supported by p≤0.001.

### Digital droplet PCR

To confirm RNA-Seq transcript expression data, we performed digital droplet (dd)PCR [64] on six randomly selected lncRNAs across three life stages. Total RNA was extracted using Trizol (ThermoFisher) from one adult *F*. *hepatica* (*n* = 3), and from the same juv21d (*n = 3*) and invitro21d (*n* = 3) RNA samples used for transcriptome sequencing as described in section 2.1. Each cDNA was generated from 500ng total RNA using the High Capacity RNA-to-cDNA kit (Thermo Fisher). Amplification for ddPCR used the EvaGreen Supermix (BioRad), including 200 nM of each primer (Supporting file 11). Data analysis used the Quantasoft package from BioRad.

### Genome localisation and expression correlation

For identification of antisense and intronic lncRNAs we used the bedtools package (v2.28; [65]. After generating bed files for transcripts, exons, introns and intergenic regions from our gtf file, we used bedtools *intersect* function to identify overlapping lncRNA and mRNA transcript loci, and sorted these by strand orientation (based on our stranded juv21d datasets) to identify antisense lncRNA:mRNA pairs. Bedtools *intersect* again allowed us to identify intronic lncRNAs by comparing lncRNA exons with mRNA introns, and again, these were sorted by strand orientation. Intergenic lncRNAs were identified as those that were not amongst our lists of antisense or intronic lncRNAs. We used the bedtools *closest* algorithm to identify the single closest upstream and downstream mRNA locus to each intergenic lncRNA locus.

Before examining expression correlation, we parsed all antisense, intronic and intergenic lncRNA:mRNA pairs to identify those pairs in which both members were DE, using a Python script for list comprehension. We then used the ‘CORREL’ function in MS Excel to calculate the correlation coefficient for lncRNA vs mRNA across TPM expression data from met, nej1h, nej3h, nej24h and juv21d libraries.

Weighted Gene Correlation Network Analysis (WGCNA) was performed using the CEMiTool R package [66].

### miRNA response element identification

To examine whether any of our lncRNAs contained binding sites for miRNAs (miRNA response elements; MRE), we employed a consensus prediction method as described by [67]. Working with the 150 miRNAs previously described in *F*. *hepatica* [9–20], we employed three miRNA target prediction tools to identify binding matches between all *F*. *hepatica* miRNAs and our lncRNAs; we retained only those lncRNA:miRNA pairs that were identified by all three algorithms. We used local instances of miRANDA [68], RNAhybrid [69](and PITA [70], accepting hits fulfilling the thresholds as used by [67]: miRanda, total score >145, energy < -10; RNAhybrid, p<0.1, energy < -22; PITA, ∆∆G < -10. LncRNA:miRNA pairs identified by all three algorithms were extracted using a custom Python script, and only these consensus pairs were accepted.

## Results

### Dataset summary

Mapping of the >1.7 bn reads (81.7% overall alignment rate; S1 Dataset) associated with 18 RNA-Seq libraries yielded 121,777 transcripts, which clustered at 90% identity into 94,137 non-redundant transcripts (Fig 1). Using BUSCO [71], we compared the quality of our assembly with the *F*. *hepatica* transcript assemblies available through WormBase Parasite WBPS14. Our dataset contained more complete BUSCOs (81.7%; PRJNA179522 = 40.4%, PRJEB25283 = 74.1%), and had fewer missing BUSCOs (12.5%; PRJNA179522 = 31.3%, PRJEB25283 = 17.9%), than previous assemblies (Fig 1).

At this point, we filtered the transcriptome to include only sequences represented by at least 100 reads, leaving a total of 63,723 supported transcripts (S2 Dataset). From this dataset, we removed 1,390 sequences with similarity to other classes of ncRNA (ribosomal RNA, transfer RNA etc) listed in the RNACentral dataset. We then extracted protein coding transcripts (containing BLAST or Pfam identities, and a complete ORF) into a counterpart dataset containing 20,930 “mRNA” transcripts (S3 Dataset), which we used to compare and examine the transcriptional and syntenic relationships between lncRNA and mRNA loci. Having removed mRNAs, we extracted our putative lncRNAs (6,436 transcripts measuring ≥200 nt and lacking significant protein coding capacity; S4, S5, S6 Dataset files).

### lncRNA and mRNA datasets are quantitatively and qualitatively distinct

**Fig 2:**
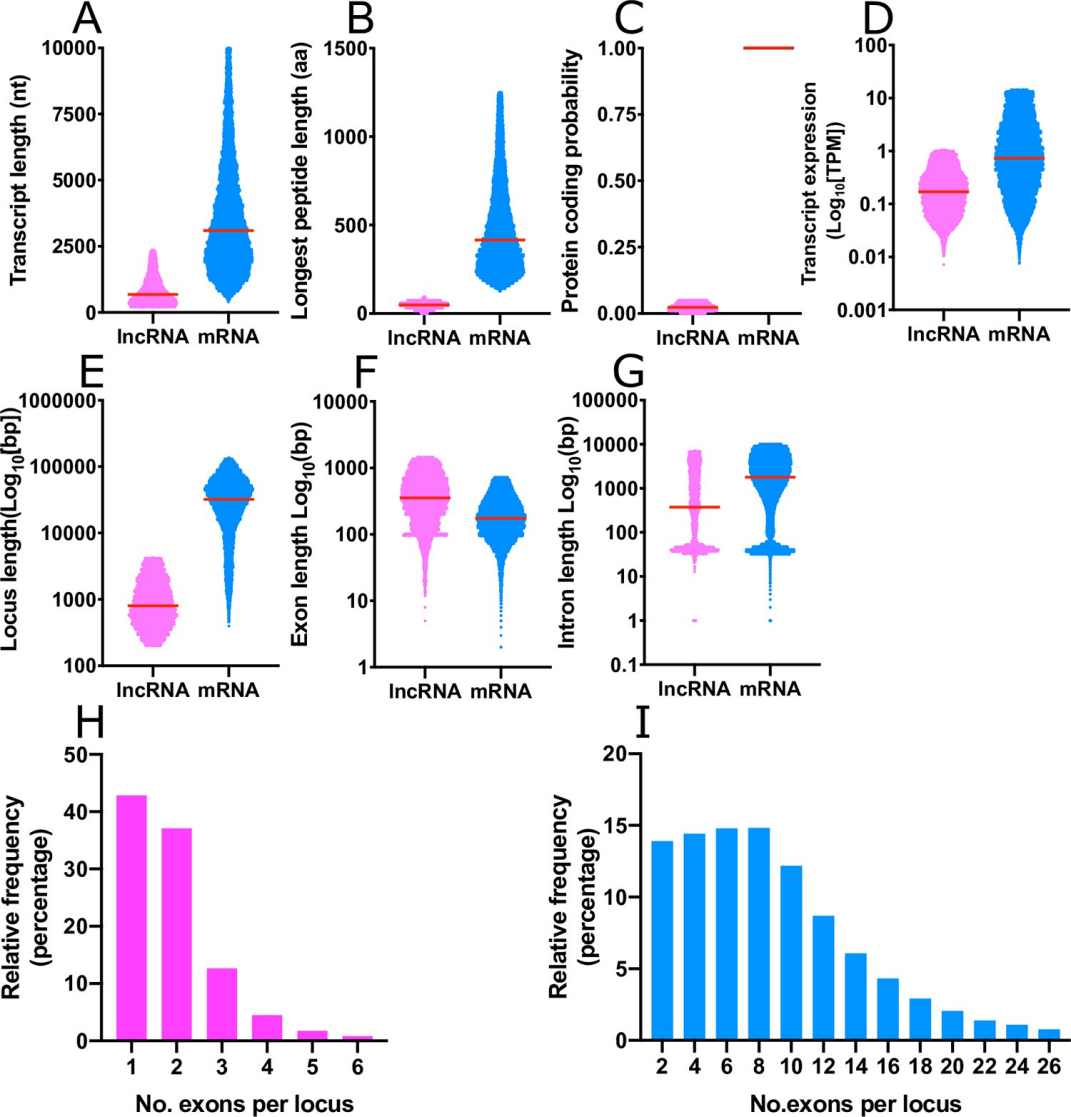
Statistical summary of mRNA and long noncoding (lnc)RNA annotations from *Fasciola hepatica*. LncRNAs and mRNAs are qualitatively and quantitatively distinct across all measures examined: (A) Transcript length; (B) Open reading frame length; (C) Probability of protein coding capacity; (D) Transcript expression/abundance; (E) Genomic locus length; (F) Exon length; (G) Intron length; and (H, I) Number of exons per locus. Each scatter graph (A-G) is composed of individual datapoints, with dataset median illustrated by a horizontal red bar. In all graphs, lncRNAs are magenta, mRNA are blue.

The pipeline described in Fig 1 identified 6,436 transcripts fitting the definition of lncRNAs, and 20,930 transcripts with protein coding capacity, which we label here as mRNAs. These datasets were distinct on all measures examined (Fig 2)–lncRNA transcripts were shorter overall (median: lncRNA = 875 nt, mRNA = 3133 nt; Fig 2A), encoded shorter open reading frames (median: lncRNA = 49 aa, mRNA = 397 aa; Fig 2B), displayed lower protein coding probability (median: lncRNA P = 0.025, mRNA P = 1.000; Fig 2C), and were expressed more scarcely (median: lncRNAs 0.122 TPM, mRNA = 0.628 TPM; Fig 2D) than mRNA transcripts. Loci coding for lncRNAs were smaller than mRNA loci (median: lncRNA = 2010 bp, mRNA = 29,261 bp; Fig 2E), with longer exons (median: lncRNA = 319 bp, mRNA = 169 bp; Fig 2F) and shorter introns (median: lncRNA = 322 bp, mRNA = 1412 bp; Fig 2G). LncRNA loci incorporated fewer exons (median = 2 exons; Fig 2H), compared to mRNA loci (median = 7 exons; Fig 2I).

### *F*. *hepatica* lncRNAs are dissimilar to lncRNAs from non-*Fasciola* species

To identify lncRNA orthologues in the closely related species, *F*. *gigantica*, we assembled an *F*. *gigantica* lncRNA dataset from available RNA-Seq datasets [23,56] using the pipeline described in Fig 1. This comprised 24,141 sequences (S7 Dataset). As described by Maciel *et al*. [45], we compared the *F*. *hepatica* and *F*. *gigantica* datasets using blastn, finding 2596 one-to-one blastn hits with an e-value cutoff of 1e-3 (S8 Dataset). We parsed these to a set of 522 *F*. *gigantica* lncRNAs matching an *F*. *hepatica* lncRNA at ≥90% sequence identity over ≥50% of the sequence length.

We also compared *F*. *hepatica* lncRNAs with published lncRNAs from other flatworms for where those sequences were openly available, using both CD-HIT and BLASTn. *F*. *hepatica* lncRNAs were compared with the >10,000 lncRNA transcripts reported from *S*. *mansoni* [41,72]. CD-HIT identified no evidence for lncRNA sequence similarity between *F*. *hepatica* and *S*. *mansoni*, while BLASTn identified a single, low-scoring orthologous pair, matching the *F*. *hepatica* lncRNA STRG.25709.1 with SmLINC02629-Ibu from *S*. *mansoni* (bitscore 56.5, E-value 4e^-07^). Likewise, BLASTn comparison with *M*. *lignano* lncRNAs [47] found no similarity, while comparisons with the RNAcentral non-coding RNA database found no similarity with the >200,000 mammalian lncRNAs within that dataset.

### lncRNA transcripts are differentially expressed during development

**Fig 3:**
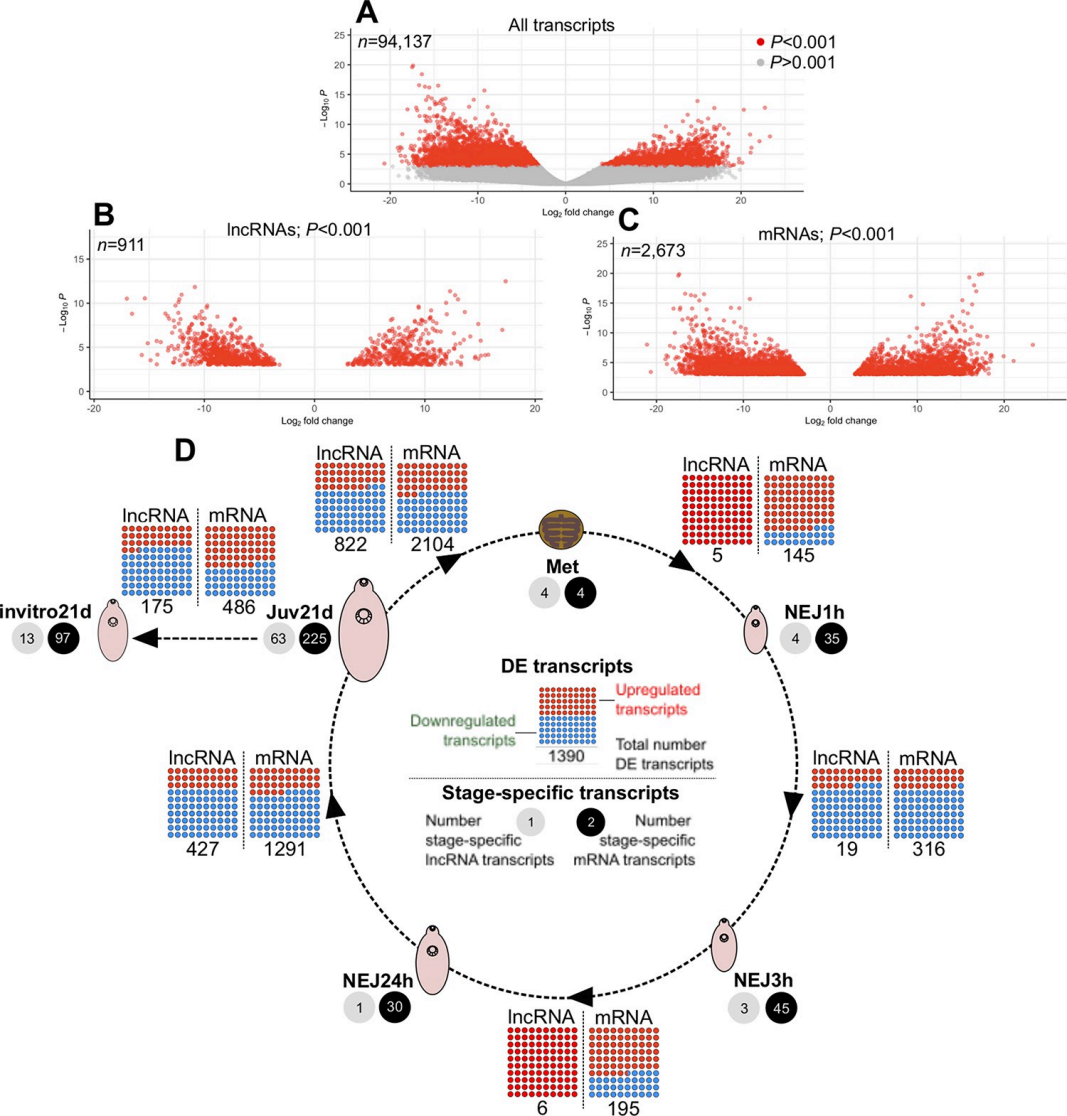
Developmental regulation of long non-coding (lnc)RNAs in intra-mammalian stages of *Fasciola hepatica*. Statistically significant differential expression (DE) was determined for lncRNA and mRNA transcripts during developmental transitions, showing that F. hepatica lncRNAs are dynamically-regulated during intra-mammalian development. A-C. Distribution of transcript fold change vs P value across all comparisons. A, all transcripts, with P-value cutoff as indicated on y-axis; B, Fold change distribution of DE lncRNAs; C, Fold change distribution of DE mRNAs. D, Summary data from DE analysis of transcripts in metacercariae (Met), newly excysted juvenile (NEJ) maintained in vitro for 1h post excystment (NEJ1h), 3h post excystment (NEJ3h) or 24h post excystment (NEJ24h), and ex vivo parasites recovered from rat livers at 21 days post infection (Juv21d). Numbers of stage-specific lncRNAs and mRNAs at each life stage are indicated, as are the numbers of DE lncRNA and mRNA transcripts, and the proportion of each upregulated or downregulated, between each developmental transition.

Differential expression (DE) analyses employed only life stages represented by at least two biological replicate libraries (met, nej1h, nej3h, nej24h, juv21d, invitro21d). Fig 3 and S9 Dataset show that all of these libraries bore stage-specific lncRNAs (met = 4; nej1h = 4; nej3h = 3; nej24h = 1; juv21d = 63; invitro21d = 13), and mRNAs (met = 42; nej1h = 35; nej3h = 45; nej24h = 30; juv21d = 225; invitro21d = 97). Using edgeR’s exact test algorithm, pairwise comparisons were performed between developmentally sequential life stages, defining DE transcripts as those with a statistically significant difference (p≤0.001) in at least one of these comparisons (Fig 3A and S10 Dataset). A total of 911 DE lncRNA transcripts (Fig 3B; 14.2% of all lncRNAs), and 2,673 DE mRNA transcripts (Fig 3C; 12.8% of all mRNAs) were identified. DE transcripts were found in every pairwise comparison (Fig 3D). To confirm these data, six randomly selected lncRNAs were tested for expression across adult, juv21d and invitro21d life stages using ddPCR; in most cases the ddPCR expression pattern correlated well with the NGS expression pattern (S11 Dataset).

### Positional relationships and expression correlations between lncRNAs and mRNAs

Based on genomic location and directionality relative to protein-coding loci, we identified three lncRNA types in *F*. *hepatica*: (i) antisense lncRNAs: expressed from a lncRNA exon on the opposite DNA strand to a protein-coding locus, and overlapping a protein-coding exon by at least 1bp; (ii) intronic lncRNAs: expressed from lncRNA exons in either orientation that reside within an intron of a protein coding locus and do not overlap an exon; (iii) intergenic lncRNAs: expressed from distinct loci that do not intersect with any protein coding locus. To delineate our lncRNAs into these categories, we used the bedtools software package, and stranded juv21d libraries (as described in Methods). Our first approach was to search for sense:antisense overlaps between lncRNA and mRNA transcripts using the bedtools *intersect* algorithm, which yielded 536 non-redundant lncRNA:mRNA transcript pairs, overlapping in opposite transcriptional orientations. The same approach identified 534 similarly oriented mRNA:mRNA pairs (i.e. transcripts in antisense orientation, where both strands encode protein), and 17 lncRNA:lncRNA pairs (i.e. transcripts in antisense orientation, where neither strand encodes protein) (S12 Dataset). In every case but one, antisense lncRNAs overlapped protein coding exons by a minimum of 94 nt, with a median overlap of 3343 nt (S12 Dataset).

Parsing the data to identify antisense pairs in which both members showed statistically significant differential expression (DE) identified 14 DE lncRNA:mRNA pairs and five DE mRNA:mRNA pairs. There were no lncRNA:lncRNA pairs in which both members were DE (S12 Dataset). For lncRNA:mRNA pairs we calculated the Pearson correlation coefficient (CC) of lncRNA vs mRNA expression (TPM) across all life stages (S12 Dataset). Accepting >0.99 and <-0.99 as cut-offs identified just seven pairs of “expression correlated” lncRNA:mRNA transcript pairs, where seven of these showed positive correlation, while two showed negative correlation.

After removing antisense transcripts from the dataset, we identified 30 intronic lncRNA:mRNA pairs (median CC = 0.407; S13 Dataset). No pairs consisted of both members as DE, and none passed the +/-0.9 CC cut-off. Finally, we identified 3795 intergenic lncRNAs where the closest transcript was an mRNA (S14 Dataset). In 29 pairs both members were DE, and only three pairs passed the +/-0.9 CC cut-off.

**Fig 4:**
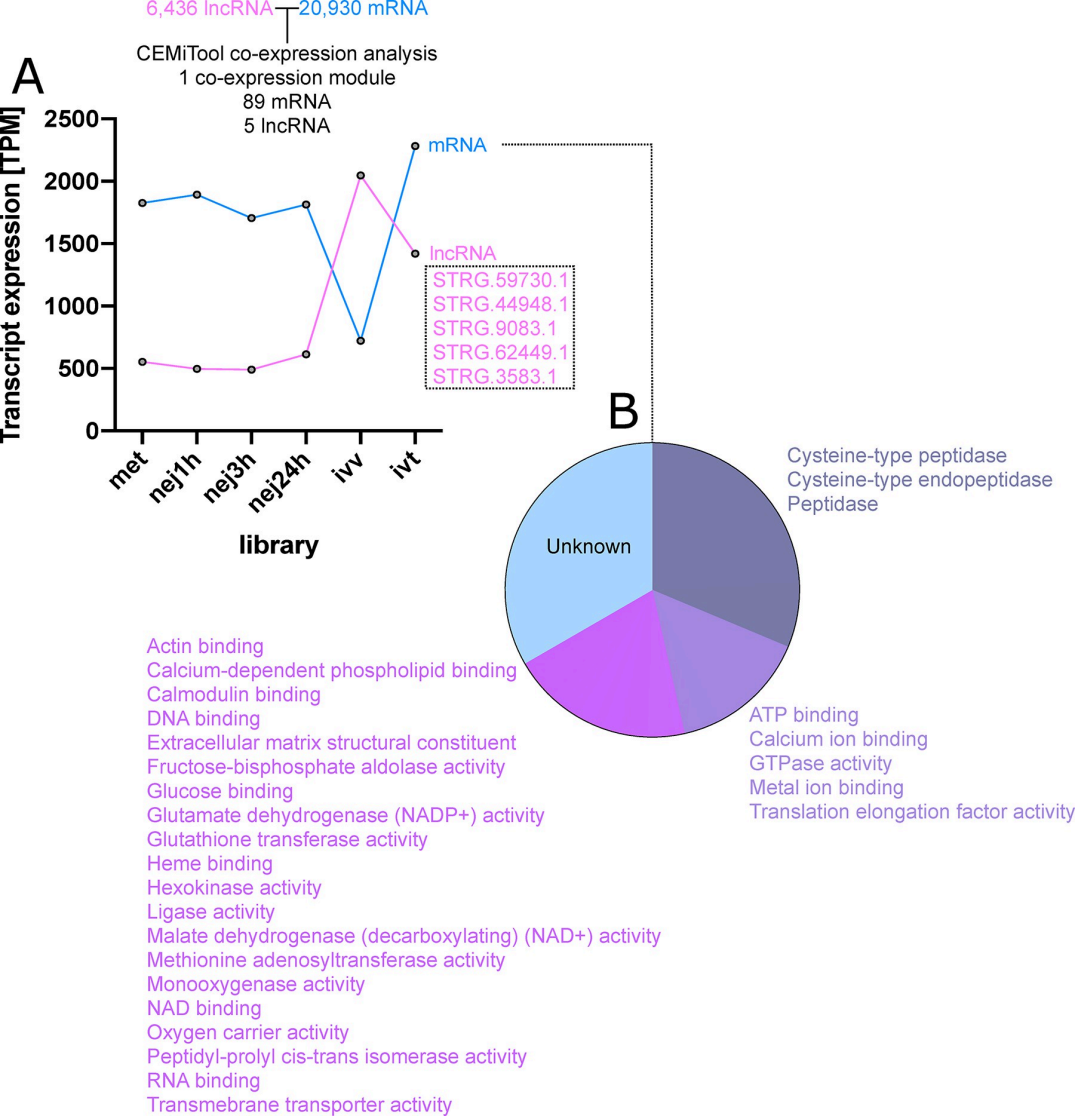
Co-expression of long noncoding (lnc)RNAs and mRNAs in *Fasciola hepatica*. (A) Global co-expression analysis of all lncRNAs and mRNAs in our dataset. CEMiTool identified one co-expression module in which five lncRNAs and 89 mRNAs show inversely correlated expression across life stage RNA-Seq libraries. (B) The 89 mRNAs comprise mostly cysteine proteases in addition to a range of other sequences as indicated. The remaining one third of matching mRNAs code for unknown sequence types that lacked BLAST homology and identifiable sequence motifs.

To explore *trans* interactions in more detail, we employed a weighted correlation network analysis approach. Here, we sought evidence for co-expression between all lncRNAs and all mRNAs across all libraries with multiple biological replicates. CEMiTool identified a single co-expression module, containing 89 mRNAs and five lncRNAs (Fig 4 and S15 Dataset). The mean TPM of the lncRNAs and mRNAs within this module were inversely correlated across longitudinally sampled libraries (Fig 4), suggesting that they may be co-regulated. Interestingly, 47% of mRNA transcripts within the module were associated with peptidase activity, including Cathepsin L and B, and legumain, potentially linking five lncRNAs with regulation of these important virulence genes.

### *F*. *hepatica* lncRNAs contain miRNA binding sites

**Fig 5:**
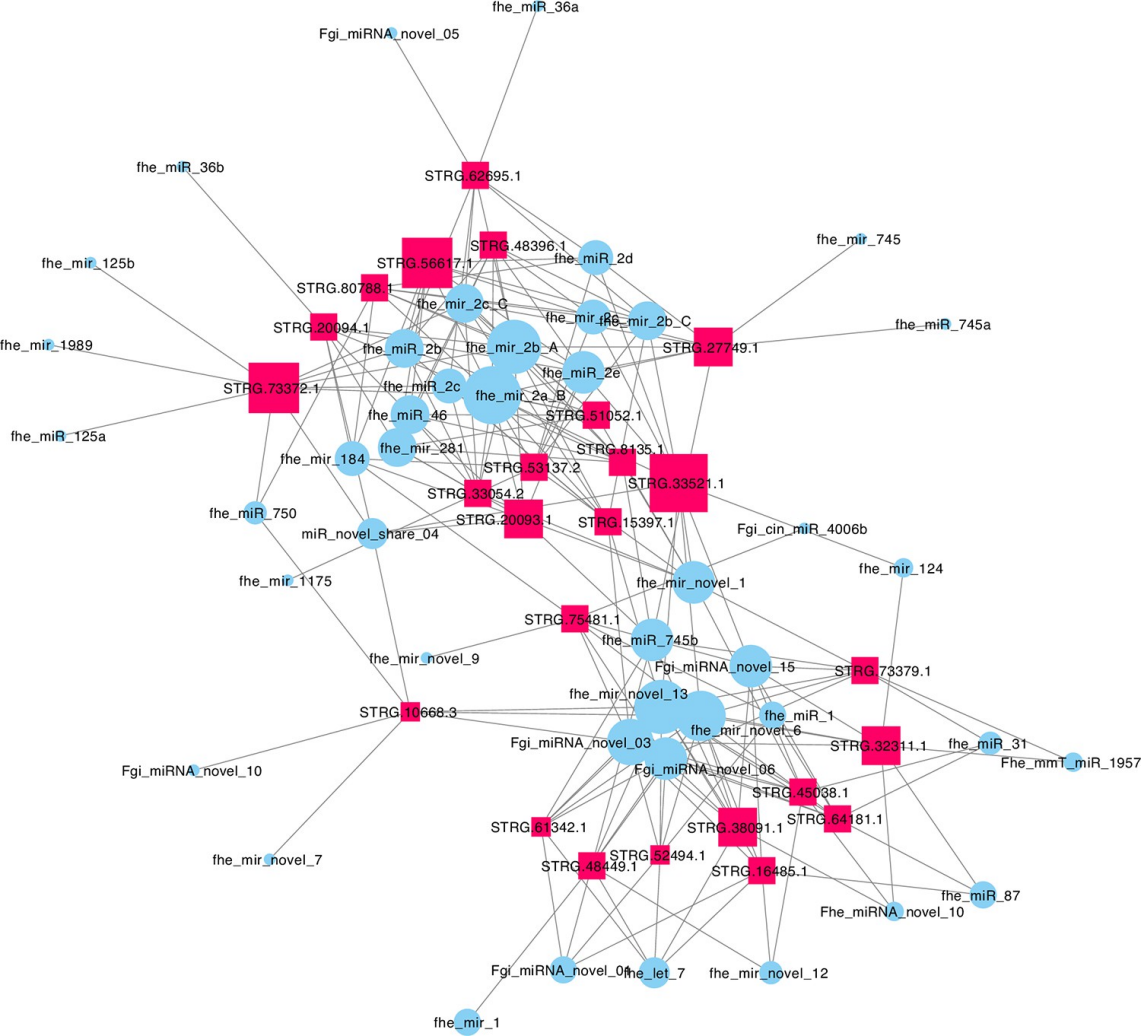
Network representation of computationally predicted binding interactions between *Fasciola hepatica* long noncoding (lnc)RNAs and micro (mi)RNAs. This network represents an excerpt of the lncRNA:miRNA network detailed in S16 Dataset, showing only the lncRNAs that matched eight or more miRNAs. LncRNA nodes are represented by red squares, miRNA nodes by blue circles. Node size is proportional to the number of connections with other nodes. Connections between nodes are represented by grey edges.

LncRNAs contain binding sites for miRNAs, implying either that they serve as “sponges” for “soaking up” individual miRNAs, or that their expression is regulated by miRNA binding and transcriptional destruction. Previous reports have identified at least 150 miRNAs in *F*. *hepatica* [9–20]. Using three miRNA binding prediction tools (miRANDA, PITA, RNAhybrid) we analysed these miRNAs in the context of binding to our lncRNAs, producing a consensus set of 4104 lncRNA:miRNA pairs that were present in outputs from all three tools (we rejected any matches that were not predicted by all three tools). S16 Dataset shows that these matches incorporated all 150 miRNAs, and 2618 lncRNAs. Individual lncRNAs contained binding sites for up to eight distinct miRNAs, with each miRNA predicted to bind up to 234 lncRNAs. Output data from PITA illustrated the number of individual binding sites per individual lncRNA:miRNA pair: while 95% of lncRNAs contained ten or fewer binding sites, 5% displayed up to 246. Fig 5 shows an excerpt of this lncRNA:miRNA network, focusing on the lncRNAs matching eight or more miRNAs. Within this network, STRG.61441.1 was the major hub lncRNA, binding to eight miRNAs. Fhe-pubnovelmiR-7 was the major hub miRNA, binding to 234 lncRNAs.

## Discussion

Fasciolosis, caused by *F*. *hepatica* and *F*. *gigantica*, is an important veterinary and zoonotic disease which requires improved diagnostic and control approaches if it is to be sustainably combatted in the long term. A key approach to identification of new control targets and diagnostic biomarkers is through increased understanding of fundamental parasite biology and host-parasite interactions. This study provides a solid foundation of data describing lncRNAs in *F*. *hepatica* for the first time, through which this need can be addressed by ongoing research. This study has performed the first classification of lncRNAs in *F*. *hepatica*, linking these to potential roles in parasite development and co-regulation of miRNAs and mRNAs. This work represents an essential precursor to functional understanding of liver fluke lncRNA biology.

Unlike other systems, *F*. *hepatica* does not yet have a unified description of the protein-coding transcriptome that would have enabled a simple subtraction-based approach to lncRNA discovery. Disparities can be seen in the varying gene complements presented by currently available studies [54,73,74]. This meant that we needed to generate a consensus transcriptome before filtering lncRNAs from mRNAs within those datasets. Our consensus transcriptome combined 18 publicly available and newly generated libraries, which together encompassed 94,137 non-redundant transcripts. This represented by far the largest transcriptome yet described for *F*. *hepatica*, with previous datasets describing 22,676 [54,73], 16,806 (WormBase ParaSite release WBPS14), or 14,462 [74] transcripts, although it is unclear whether those previously published datasets retained non-coding sequences. BUSCO metrics confirmed that our transcriptome assembly was the most complete yet published for *Fasciola*, giving us confidence in proceeding to lncRNA discovery. This transcriptome was then filtered to include only sequences represented by at least 100 reads (an arbitrary selection which aimed to balance well-supported transcript models while maintaining adequate detection of rare transcripts), followed by removal of irrelevant classes of ncRNA, and protein coding transcripts. Our final pool of putative lncRNAs represented 6,436 transcripts measuring ≥200 nt and lacking significant protein coding capacity. A separate pool of protein-coding transcripts contained 20,930 mRNAs.

To identify lncRNA transcripts that we may have mis-annotated, or those from published datasets that may have been erroneously annotated as protein-coding, we used BLAST to compare all lncRNAs with previously published *F*. *hepatica* transcript annotations. BLASTx did not identify any identical matches between our lncRNAs and previously predicted proteins. BLASTn analyses identified 37 lncRNAs matching 20 sequences previously annotated as mRNAs. However, we found that these matching sequences had either no identifiable protein sequence characteristics and/or were classed as hypothetical proteins. We therefore retained these in our lncRNA dataset as putative lncRNAs.

Previous literature from other species showed that lncRNAs were distinct from mRNAs across a wide range of measures [37,75–77]. We confirmed that these findings also applied to our dataset, showing that lncRNAs were shorter than mRNAs in gene locus length, overall transcript length and open reading frame length. LncRNA loci also tended to display fewer, longer exons and shorter introns than mRNA loci. LncRNAs were also expressed more scarcely than mRNA transcripts. Inter-species comparisons of nucleic acid sequences can provide insights into evolution, with sequence conservation potentially indicating functional conservation. We used public datasets and our pipeline to identify lncRNAs in *F*. *gigantica*. Comparison with *F*. *hepatica* lncRNAs identified at least 522 orthologues (defined as those showing ≥ 90% sequence identity across ≥50% of the aligned sequence). This suggests common evolutionary origin of lncRNAs between these two species although the majority of lncRNAs in both species appeared to be species specific.

There are currently limited opportunities to make this comparison for lncRNAs with other flatworms, with *S*. *mansoni*, *S*. *japonicum*, *E*. *granulosus* and *M*. *lignano* the only other flatworm lncRNA datasets currently available [41–47]. In comparing *F*. *hepatica* lncRNAs with these datasets we found an almost complete lack of lncRNA sequence conservation. This absence of primary sequence similarity between genera is not surprising and suggests that, like in other organisms, the rapid evolution of lncRNAs invalidates primary sequence similarity as a means of identifying lncRNA homologs [78–80]. Similarly, BLASTn comparisons with the RNAcentral non-coding RNA database found no similarity between our lncRNAs and the >200,000 mammalian lncRNAs within that dataset. This distinction between host and parasite lncRNA sequences could provide a source of new diagnostic biomarkers, if future work can show that lncRNAs are secreted by fluke or other helminths, as is the case for miRNAs [81].

Rather than primary sequence similarity, lncRNAs are thought to be conserved between species according to spatiotemporal and syntenic locus expression [82]. Indeed, Maciel et al. [45] showed for the first time that synteny conservation can be used for lncRNA discovery in flatworms, where 14% of *S*. *japonicum* intergenic lncRNAs had conserved synteny with those in *S*. *mansoni*. Other studies have also shown lncRNA sequence conservation between *S*. *mansoni*, *S*. *japonicum* and *S*. *haematobium*, albeit with lower similarity than that seen in mRNA comparisons, and using small sample sizes and an older version of the *S*. *mansoni* genome [42]. Syntenic comparisons have not yet been performed between flatworm genera, but undoubtedly represent a necessary future direction for lncRNA discovery in flatworms.

Differential expression (DE) analyses employed only life stages represented by at least two biological replicate libraries (met, nej1h, nej3h, nej24h, juv21d, invitro21d). All these libraries bore stage-specific lncRNAs and mRNAs, with 911 DE lncRNA transcripts and 2,673 DE mRNA transcripts identified in pairwise comparisons between sequential lifestages. DE transcripts were found in all pairwise comparisons, with the number of DE transcripts roughly proportional to the time-course length between stages–the fewest between met:nej1h (5 lncRNA, 145 mRNA), and the most between juv21d:met (822 lncRNA, 2104 mRNA). Comparison of juv21d with invitro21d samples (respectively, flukes recovered from rat livers 21 days after infection, or maintained *in vitro* for 21 days [19,83], demonstrated the presence of 13 lncRNAs found uniquely in invitro21d samples, with 63 found uniquely in juv21d worms. Given the key developmental differences between these groups [19], these lncRNAs have potential importance for fluke development, and represent priority targets for functional genomics experiments. The strikingly similar proportions of DE lncRNAs and mRNAs (respectively, 14.2% and 12.8%), shows that lncRNAs are at least as transcriptionally dynamic as coding RNAs, and supports the hypothesis that they have essential and stage-specific functions during the *F*. *hepatica* intra-mammalian life cycle.

While our focus was not to reannotate the mRNA transcriptome of *F*. *hepatica*, our lncRNA annotation method did also generate protein-coding mRNAs and associated DE data. Briefly, the most highly regulated mRNA transcripts (≥16 fold) in our dataset closely reflect previous observations [54,73], and include sequences within GO processes describing cell adhesion, cell division, glycolysis, metal ion homeostasis, muscle function, proteolysis, protein synthesis and modification, RNA transcriptional control and signal transduction.

The purpose of these data was to identify and explore potential interactions between lncRNAs and mRNAs. Various classification schemes exist for lncRNAs, but one of the most commonly used groups sequences based on their genomic location and directionality relative to protein-coding loci [82]. According to this classification scheme, we used a subtractive approach to identify antisense, intronic and intergenic lncRNA types in *F*. *hepatica*. These classifications are important because they can inform potential interactions with protein coding genes, for example *cis* natural antisense transcripts (NATs) and intronic lncRNAs can affect the expression of corresponding sense transcripts from their “host” gene, while *trans*-antisense and intergenic lncRNAs can impact expression of distant genomic loci. Our searches yielded 536 antisense overlapping lncRNA and mRNA transcript pairs (where the mRNA was considered “sense”). These data also identified 534 complementary mRNA:mRNA pairs, and 17 complementary lncRNA:lncRNA pairs. These data show that lncRNA and/or mRNA loci may overlap on the genome, to produce pairs of antisense/complementary-oriented transcripts. The mRNA:mRNA pairings in particular are relevant to RNA interference (RNAi) experiments, where design of double stranded (ds)RNA triggers to a target gene on one of these strands could lead to unintended off-target effects on the opposite-strand gene.

Given evidence that lncRNAs can interact transcriptionally in *cis* or *trans* fashion with mRNAs, we looked for evidence of transcriptional interaction between antisense transcripts. For example, data from human cell lines show that antisense lncRNAs can regulate the expression of the mRNAs with which they overlap [30,31]. We reasoned that correlated expression might be most clearly identified in cases where both members of an antisense pair were DE. Parsing the data to identify pairs in which this was the case identified 14 DE lncRNA:mRNA pairs. We also found five DE mRNA:mRNA pairs, but no lncRNA:lncRNA pairs in which both members were DE. In case these criteria were prohibitively restrictive, we also calculated the Pearson correlation coefficient (CC) of lncRNA vs mRNA expression (TPM) for all 536 pairs across all life stages. Accepting >0.99 and <-0.99 as cut-offs identified just 53 pairs of DE lncRNA/mRNA transcript pairs that were highly correlated. These similar data suggested that correlated expression between *cis* oriented antisense transcripts might not be a widespread phenomenon, but we cannot rule out the possibility that some antisense lncRNA regulation of *cis* mRNA transcripts may occur. Functional genomics experiments will be necessary to test hypotheses around linkages between individual transcripts, and separate interacting transcripts from physically overlapping transcripts that do not interact [84–87]. As well as informing studies on evolution of the *F*. *hepatica* genome, these data have important practical implications in avoiding off-target effects in RNAi or CRISPR/Cas9 experimental design.

After removing these antisense transcripts from the dataset, we used similar methods to identify 30 intronic lncRNA:mRNA pairs (i.e. lncRNAs located within an mRNA intron in either orientation). No pair consisted of both members as DE, and none passed the +/-0.9 CC cut-off. Finally, we identified 3795 intergenic lncRNAs where the closest transcript was an mRNA (Supporting file 12). In 29 pairs both members were DE, and only three pairs passed the +/-0.9 CC cut-off. Further exploration of these data could focus on intronic lncRNAs as a potential source of long interspersed element (LINE) type transposable elements (TEs).

These findings differ from those from other systems, where lncRNAs have been shown to affect transcription of antisense overlapping and neighbouring protein-coding genes [30,31,88]. This prompted us to explore *trans* lncRNA interactions in more detail, where lncRNAs may regulate distant mRNA loci through epigenetic interaction [89]. We approached this using weighted correlation network analysis, via the CEMiTool R package, focusing broadly on co-expression between all lncRNAs and all mRNAs across all libraries in our datasets. This identified a single co-expression module, containing 89 mRNAs and five lncRNAs, which showed inversely correlated expression suggesting interaction and/or co-regulation. Interestingly, 47% of mRNA transcripts within the module were associated with peptidase activity, including Cathepsin L and B, and legumain (Fig 4). This is unsurprising given the preponderance of proteases amongst the most highly regulated *F*. *hepatica* transcripts [54,73]; these data represent the first link between lncRNAs and potential co-regulation of protease expression in *F*. *hepatica*. Future experiments should cement this link by silencing one or more of the lncRNAs and assaying for fluctuation in linked protease transcripts. Interrupting this regulatory interaction with lncRNA-targeting, oligonucleotide-based drugs [50,51], could represent a route towards new therapeutics for liver fluke infections. This idea is supported by the existence of licensed nucleic-acid-based drugs for several human conditions. Antisense oligonucleotide therapeutics, of the type that could be developed to target lncRNAs, include fomivirsen, which was used to treat cytomegalovirus retinitis [90] before being superceded by newer therapies, tofersen, which inhibits translation of superoxide dismutase in amytrophic lateral sclerosis [91] and eteplirsen, which causes exon skipping in the dystrophin transcript, used in therapy of Duchenne muscular dystrophy [92].

One of the most widespread hypotheses for lncRNA function is the competing endogenous (ce)RNA hypothesis, which holds that lncRNAs contain miRNA binding sites (known as miRNA Response Elements, or MREs), enabling them to act as ‘sponges’ for miRNAs. This ‘sponging’ phenomenon is thought to promote competition for miRNA binding with cognate mRNA targets, enabling fine control of miRNA regulation of mRNA target transcripts [93,94]. Alternatively, the presence of miRNA binding sites may simply indicate that lncRNAs are transcriptionally regulated by miRNA binding in the same manner as mRNAs [95]. Since both possibilities could yield important insights into liver fluke lncRNA biology, we used in silico tools to explore the presence of miRNA binding sites on lncRNAs.

One hundred and fifty miRNAs have been previously reported in *F*. *hepatica* [9–20]. We generated in silico binding predictions between these miRNAs and our lncRNAs, producing a consensus set of 4104 lncRNA:miRNA pairs. These matches incorporated all 150 miRNAs, and 2618 lncRNAs. Individual lncRNAs contained binding sites for up to eight distinct miRNAs, with each miRNA predicted to bind up to 234 lncRNAs. Output data from PITA illustrated the number of individual binding sites per individual lncRNA:miRNA pair: while 95% of lncRNAs contained ten or fewer binding sites, the remaining 5% displayed up to 246. Network analysis identified STRG.61441.1 as the major hub lncRNA, binding to eight miRNAs. Fhe-pubnovelmiR-7 was the major hub miRNA, binding to 234 lncRNAs. These data support the possibility of miRNA-lncRNA interactions, that might manifest as either traditional miRNA-driven post-transcriptional regulation of lncRNA expression, or a ceRNA function for the described miRNAs. Experimental evidence will be required to test these hypotheses further. If supported, these could provide an additional route to new therapies similar to those described for lncRNAs above; indeed miRNA inhibiting drugs are in clinical trials for many conditions including haemophilia, hepatitis B, hypercholesterolaemia, nonalcoholic fatty liver disease and various cancers [96], suggesting the possibility of translating these approaches into the anthelmintic sphere.

This study has profiled the first set of lncRNAs in the liver fluke, *F*. *hepatica*. These non-coding RNAs were expressed across multiple intra-mammalian developmental stages, showing dynamic regulation between life-stages that suggests life-stage specific functions. *In silico* analyses supported important roles for lncRNAs in transcriptional regulation including: (i) an inverse correlation of lncRNA expression with mRNAs, suggesting co-regulation of these sequences, and; (ii) the widespread location of miRNA binding sites on lncRNAs, suggesting miRNA regulation of lncRNA, or vice versa. These data represent a steppingstone towards an understanding of non-coding RNA biology in *F*. *hepatica*, an area which remains poorly understood across eukaryotes but could expose new therapeutic and diagnostic options for parasite infections globally.

## Supporting information

S1 Dataset Hisat2 alignment statistics of Illumina RNA-Seq reads during mapping to *Fasciola hepatica* genome (PRJEB25283, WBPS14). (DOCX)

S2 Dataset Mapped assembled transcript sequences each supported by at least 100 RNA-Seq reads. Entire dataset.
(ZIP)

S3 Dataset Mapped assembled transcript sequences each supported by at least 100 RNA-Seq reads. Protein-coding sequences only.
(TXT)

S4 Dataset Mapped assembled transcript sequences each supported by at least 100 RNA-Seq reads. Long non-coding RNA sequences only.
(TXT)

S5 Dataset BLASTx comparison of predicted long non-coding RNA sequences with predicted proteins from the *Fasciola hepatica* genome (WBPS14 PRJEB25283). (XLSX)

S6 Dataset BLASTn comparison of predicted long non-coding RNA sequences with mRNA transcripts from the *Fasciola hepatica* genome (WBPS14 PRJEB25283). (XLSX)

S7 Dataset BLASTn comparison of *Fasciola hepatica* long non-coding RNA sequences with a *Fasciola gigantica* transcriptome (Davey et al., 2022) [56]. (TXT)

S8 Dataset Long non-coding RNA sequences identified from a *Fasciola gigantica* transcriptome (Davey et al., 2022) [56]. (XLSX)

S9 Dataset Long non-coding RNAs and mRNAs expressed in only one *Fasciola hepatica* life stage. (XLSX)

S10 Dataset Differentially expressed long non-coding RNAs and mRNAs in comparisons between sequential developmental stages. (XLSX)

S11 Dataset Comparison of expression level of six long non-coding (lnc)RNAs measured with next generation sequencing (NGS) (blue), and digital droplet (dd)PCR (red). Each lncRNA was measured across three life-stage libraries: Adult *Fasciola hepatica*, 21 day in vitro juvenile *F*. *hepatica* (ivt) and 21 day in vivo juvenile *F*. *hepatica* (ivv). Each datapoint represents the mean±SEM of at least three biological replicates. Under each graph, the primer sets used for ddPCR amplification are indicated.
(PDF)

S12 Dataset Overlapping antisense oriented long non-coding RNA vs mRNA pairs. Note that there are three tabs comparing: 1. Antisense lncRNA vs mRNA; 2. Antisense mRNA vs mRNA; 3. Antisense lncRNA vs lncRNA.
(XLSX)

S13 Dataset Intronic long non-coding RNA vs mRNA pairs. (XLSX)

S14 Dataset Intergenic long non-coding RNA vs mRNA pairs. (XLSX)

S15 Dataset Co-expression module containing long non-coding RNA and mRNA transcripts. (XLSX)

S16 Dataset Consensus predictions of interactions between Fasciola hepatica long non-coding RNA and microRNA sequences. (XLSX)
