# Supplementary material for: Discovery of long non-coding RNAs in the liver fluke, Fasciola hepatica
Source: PLoS Negl Trop Dis. 2023 Sep 28;17(9):e0011663. doi: 10.1371/journal.pntd.0011663 (PMC10564125; doi:10.1371/journal.pntd.0011663)
Supplement: S1 Dataset — (DOCX) [file pntd.0011663.s001.docx]

**# S1 Dataset. RNA-Seq LIBRARY IDs AND HISAT2 ALIGNMENT STATISTICS OF ILLUMINA RNA-SEQ READS DURING MAPPING TO THE FASCIOLA HEPATICA GENOME (PRJEB25283, WBPS14)**

***F. hepatica* RNA-Seq LIBRARIES USED**

**STUDY PRJEB49655**

ftp.sra.ebi.ac.uk/vol1/run/ERR103/ERR10320286/2-21d_in_vivo_2_170224_L001_R2.fastq.gz

ftp.sra.ebi.ac.uk/vol1/run/ERR103/ERR10314408/2-21d_in_vivo_2_170213_L008_R1.fastq.gz

ftp.sra.ebi.ac.uk/vol1/run/ERR103/ERR10320357/3-21d_in_vivo_3_170224_L001_R2.fastq.gz

ftp.sra.ebi.ac.uk/vol1/run/ERR917/ERR9171385/4-21d_in_vitro_1_170213_L006_R1.fastq.gz

ftp.sra.ebi.ac.uk/vol1/run/ERR924/ERR9246635/5-21d_in_vitro_2_170213_L006_R1.fastq.gz

ftp.sra.ebi.ac.uk/vol1/run/ERR924/ERR9247141/5-21d_in_vitro_2_170213_L008_R1.fastq.gz

ftp.sra.ebi.ac.uk/vol1/run/ERR924/ERR9248464/6-21d_in_vitro_3_170213_L006_R1.fastq.gz

ftp.sra.ebi.ac.uk/vol1/run/ERR924/ERR9248650/6-21d_in_vitro_3_170213_L008_R2.fastq.gz

ftp.sra.ebi.ac.uk/vol1/run/ERR924/ERR9248653/6-21d_in_vitro_3_170224_L001_R1.fastq.gz

ftp.sra.ebi.ac.uk/vol1/run/ERR953/ERR9539108/1-21d_in_vivo_1_170213_L008_R1.fastq.gz

ftp.sra.ebi.ac.uk/vol1/run/ERR922/ERR9228072/4-21d_in_vitro_1_170213_L008_R2.fastq.gz

ftp.sra.ebi.ac.uk/vol1/run/ERR103/ERR10314292/2-21d_in_vivo_2_170213_L006_R1.fastq.gz

ftp.sra.ebi.ac.uk/vol1/run/ERR103/ERR10320287/2-21d_in_vivo_2_170224_L001_R1.fastq.gz

ftp.sra.ebi.ac.uk/vol1/run/ERR103/ERR10313047/1-21d_in_vivo_1_170224_L001_R1.fastq.gz

ftp.sra.ebi.ac.uk/vol1/run/ERR103/ERR10320356/3-21d_in_vivo_3_170224_L001_R1.fastq.gz

ftp.sra.ebi.ac.uk/vol1/run/ERR917/ERR9178030/4-21d_in_vitro_1_170213_L006_R2.fastq.gz

ftp.sra.ebi.ac.uk/vol1/run/ERR953/ERR9539054/1-21d_in_vivo_1_170213_L006_R1.fastq.gz

ftp.sra.ebi.ac.uk/vol1/run/ERR953/ERR9539061/1-21d_in_vivo_1_170213_L006_R2.fastq.gz

ftp.sra.ebi.ac.uk/vol1/run/ERR923/ERR9230605/4-21d_in_vitro_1_170224_L001_R2.fastq.gz

ftp.sra.ebi.ac.uk/vol1/run/ERR924/ERR9246823/5-21d_in_vitro_2_170213_L006_R2.fastq.gz

ftp.sra.ebi.ac.uk/vol1/run/ERR924/ERR9248312/5-21d_in_vitro_2_170213_L008_R2.fastq.gz

ftp.sra.ebi.ac.uk/vol1/run/ERR924/ERR9248377/5-21d_in_vitro_2_170224_L001_R1.fastq.gz

ftp.sra.ebi.ac.uk/vol1/run/ERR924/ERR9248462/5-21d_in_vitro_2_170224_L001_R2.fastq.gz

ftp.sra.ebi.ac.uk/vol1/run/ERR924/ERR9248466/6-21d_in_vitro_3_170213_L006_R2.fastq.gz

ftp.sra.ebi.ac.uk/vol1/run/ERR924/ERR9248647/6-21d_in_vitro_3_170213_L008_R1.fastq.gz

ftp.sra.ebi.ac.uk/vol1/run/ERR924/ERR9248655/6-21d_in_vitro_3_170224_L001_R2.fastq.gz

ftp.sra.ebi.ac.uk/vol1/run/ERR103/ERR10313925/1-21d_in_vivo_1_170224_L001_R2.fastq.gz

ftp.sra.ebi.ac.uk/vol1/run/ERR103/ERR10314407/2-21d_in_vivo_2_170213_L006_R2.fastq.gz

ftp.sra.ebi.ac.uk/vol1/run/ERR103/ERR10320288/3-21d_in_vivo_3_170213_L006_R1.fastq.gz

ftp.sra.ebi.ac.uk/vol1/run/ERR103/ERR10320353/3-21d_in_vivo_3_170213_L006_R2.fastq.gz

ftp.sra.ebi.ac.uk/vol1/run/ERR103/ERR10320354/3-21d_in_vivo_3_170213_L008_R1.fastq.gz

ftp.sra.ebi.ac.uk/vol1/run/ERR103/ERR10320355/3-21d_in_vivo_3_170213_L008_R2.fastq.gz

ftp.sra.ebi.ac.uk/vol1/run/ERR103/ERR10321333/2-21d_in_vivo_2_170213_L008_R2.fastq.gz

ftp.sra.ebi.ac.uk/vol1/run/ERR918/ERR9185329/4-21d_in_vitro_1_170213_L008_R1.fastq.gz

ftp.sra.ebi.ac.uk/vol1/run/ERR922/ERR9229209/4-21d_in_vitro_1_170224_L001_R1.fastq.gz

**STUDY PRJEB6904**

ftp.sra.ebi.ac.uk/vol1/run/ERR576/ERR576960/7_Index_25.NEJ1hrExcystedPM_R1.fastq.gz

ftp.sra.ebi.ac.uk/vol1/run/ERR576/ERR576961/8_Index_25.NEJ1hrExcystedPM_R1.fastq.gz

ftp.sra.ebi.ac.uk/vol1/run/ERR576/ERR576953/8_Index_14.Adult6-8weeksPM_R1.fastq.gz

ftp.sra.ebi.ac.uk/vol1/run/ERR576/ERR576957/8_Index_21.Juvenile21daysMR1_R1.fastq.gz

ftp.sra.ebi.ac.uk/vol1/run/ERR576/ERR576958/7_Index_23.Metacercariaedormant0hrPM_R1.fastq.gz

ftp.sra.ebi.ac.uk/vol1/run/ERR576/ERR576962/7_Index_27.NEJ3hrsExcystedPM_R1.fastq.gz

ftp.sra.ebi.ac.uk/vol1/run/ERR576/ERR576966/HI.0658.008.Index_6.NEJ_1hr_Dalton_R1.fastq.gz

ftp.sra.ebi.ac.uk/vol1/run/ERR576/ERR576952/7_Index_14.Adult6-8weeksPM_R1.fastq.gz

ftp.sra.ebi.ac.uk/vol1/run/ERR576/ERR576963/8_Index_27.NEJ3hrsExcystedPM_R1.fastq.gz

ftp.sra.ebi.ac.uk/vol1/run/ERR576/ERR576969/HI.0661.008.Index_7.NEJ_3hr_Dalton_R1.fastq.gz

ftp.sra.ebi.ac.uk/vol1/run/ERR576/ERR576964/HI.0658.008.Index_1.Mets_0hr-b_Dalton_R1.fastq.gz

ftp.sra.ebi.ac.uk/vol1/run/ERR576/ERR576956/7_Index_21.Juvenile21daysMR1_R1.fastq.gz

ftp.sra.ebi.ac.uk/vol1/run/ERR576/ERR576965/HI.0658.008.Index_2.Mets_0hr_Dalton_R1.fastq.gz

ftp.sra.ebi.ac.uk/vol1/run/ERR576/ERR576967/HI.0661.008.Index_3.NEJ_24hr-b_Dalton_R1.fastq.gz

ftp.sra.ebi.ac.uk/vol1/run/ERR576/ERR576954/7_Index_20.Eggafter8weeksPT_R1.fastq.gz

ftp.sra.ebi.ac.uk/vol1/run/ERR576/ERR576955/8_Index_20.Eggafter8weeksPT_R1.fastq.gz

ftp.sra.ebi.ac.uk/vol1/run/ERR576/ERR576959/8_Index_23.Metacercariaedormant0hrPM_R1.fastq.gz

ftp.sra.ebi.ac.uk/vol1/run/ERR576/ERR576968/HI.0661.008.Index_5.NEJ_24hr_Dalton_R1.fastq.gz

ftp.sra.ebi.ac.uk/vol1/run/ERR576/ERR576960/7_Index_25.NEJ1hrExcystedPM_R2.fastq.gz

ftp.sra.ebi.ac.uk/vol1/run/ERR576/ERR576961/8_Index_25.NEJ1hrExcystedPM_R2.fastq.gz

ftp.sra.ebi.ac.uk/vol1/run/ERR576/ERR576953/8_Index_14.Adult6-8weeksPM_R2.fastq.gz

ftp.sra.ebi.ac.uk/vol1/run/ERR576/ERR576957/8_Index_21.Juvenile21daysMR1_R2.fastq.gz

ftp.sra.ebi.ac.uk/vol1/run/ERR576/ERR576958/7_Index_23.Metacercariaedormant0hrPM_R2.fastq.gz

ftp.sra.ebi.ac.uk/vol1/run/ERR576/ERR576962/7_Index_27.NEJ3hrsExcystedPM_R2.fastq.gz

ftp.sra.ebi.ac.uk/vol1/run/ERR576/ERR576966/HI.0658.008.Index_6.NEJ_1hr_Dalton_R2.fastq.gz

ftp.sra.ebi.ac.uk/vol1/run/ERR576/ERR576952/7_Index_14.Adult6-8weeksPM_R2.fastq.gz

ftp.sra.ebi.ac.uk/vol1/run/ERR576/ERR576963/8_Index_27.NEJ3hrsExcystedPM_R2.fastq.gz

ftp.sra.ebi.ac.uk/vol1/run/ERR576/ERR576969/HI.0661.008.Index_7.NEJ_3hr_Dalton_R2.fastq.gz

ftp.sra.ebi.ac.uk/vol1/run/ERR576/ERR576964/HI.0658.008.Index_1.Mets_0hr-b_Dalton_R2.fastq.gz

ftp.sra.ebi.ac.uk/vol1/run/ERR576/ERR576956/7_Index_21.Juvenile21daysMR1_R2.fastq.gz

ftp.sra.ebi.ac.uk/vol1/run/ERR576/ERR576965/HI.0658.008.Index_2.Mets_0hr_Dalton_R2.fastq.gz

ftp.sra.ebi.ac.uk/vol1/run/ERR576/ERR576967/HI.0661.008.Index_3.NEJ_24hr-b_Dalton_R2.fastq.gz

ftp.sra.ebi.ac.uk/vol1/run/ERR576/ERR576954/7_Index_20.Eggafter8weeksPT_R2.fastq.gz

ftp.sra.ebi.ac.uk/vol1/run/ERR576/ERR576955/8_Index_20.Eggafter8weeksPT_R2.fastq.gz

ftp.sra.ebi.ac.uk/vol1/run/ERR576/ERR576959/8_Index_23.Metacercariaedormant0hrPM_R2.fastq.gz

ftp.sra.ebi.ac.uk/vol1/run/ERR576/ERR576968/HI.0661.008.Index_5.NEJ_24hr_Dalton_R2.fastq.gz

***F. gigantica* RNA-Seq LIBRARIES USED**

**STUDY PRJNA350370**

ftp.sra.ebi.ac.uk/vol1/fastq/SRR133/066/SRR13334766/SRR13334766_1.fastq.gz

ftp.sra.ebi.ac.uk/vol1/fastq/SRR133/069/SRR13334769/SRR13334769_1.fastq.gz

ftp.sra.ebi.ac.uk/vol1/fastq/SRR133/070/SRR13334770/SRR13334770_1.fastq.gz

ftp.sra.ebi.ac.uk/vol1/fastq/SRR133/073/SRR13334773/SRR13334773_1.fastq.gz

ftp.sra.ebi.ac.uk/vol1/fastq/SRR133/074/SRR13334774/SRR13334774_1.fastq.gz

ftp.sra.ebi.ac.uk/vol1/fastq/SRR133/075/SRR13334775/SRR13334775_1.fastq.gz

ftp.sra.ebi.ac.uk/vol1/fastq/SRR133/078/SRR13334778/SRR13334778_1.fastq.gz

ftp.sra.ebi.ac.uk/vol1/fastq/SRR133/082/SRR13334782/SRR13334782_1.fastq.gz

ftp.sra.ebi.ac.uk/vol1/fastq/SRR133/083/SRR13334783/SRR13334783_1.fastq.gz

ftp.sra.ebi.ac.uk/vol1/fastq/SRR133/084/SRR13334784/SRR13334784_1.fastq.gz

ftp.sra.ebi.ac.uk/vol1/fastq/SRR133/086/SRR13334786/SRR13334786_1.fastq.gz

ftp.sra.ebi.ac.uk/vol1/fastq/SRR133/087/SRR13334787/SRR13334787_1.fastq.gz

ftp.sra.ebi.ac.uk/vol1/fastq/SRR133/068/SRR13334768/SRR13334768_1.fastq.gz

ftp.sra.ebi.ac.uk/vol1/fastq/SRR133/072/SRR13334772/SRR13334772_1.fastq.gz

ftp.sra.ebi.ac.uk/vol1/fastq/SRR133/077/SRR13334777/SRR13334777_1.fastq.gz

ftp.sra.ebi.ac.uk/vol1/fastq/SRR133/080/SRR13334780/SRR13334780_1.fastq.gz

ftp.sra.ebi.ac.uk/vol1/fastq/SRR133/081/SRR13334781/SRR13334781_1.fastq.gz

ftp.sra.ebi.ac.uk/vol1/fastq/SRR133/085/SRR13334785/SRR13334785_1.fastq.gz

ftp.sra.ebi.ac.uk/vol1/fastq/SRR133/089/SRR13334789/SRR13334789_1.fastq.gz

ftp.sra.ebi.ac.uk/vol1/fastq/SRR133/065/SRR13334765/SRR13334765_1.fastq.gz

ftp.sra.ebi.ac.uk/vol1/fastq/SRR133/067/SRR13334767/SRR13334767_1.fastq.gz

ftp.sra.ebi.ac.uk/vol1/fastq/SRR133/071/SRR13334771/SRR13334771_1.fastq.gz

ftp.sra.ebi.ac.uk/vol1/fastq/SRR133/076/SRR13334776/SRR13334776_1.fastq.gz

ftp.sra.ebi.ac.uk/vol1/fastq/SRR133/079/SRR13334779/SRR13334779_1.fastq.gz

ftp.sra.ebi.ac.uk/vol1/fastq/SRR133/066/SRR13334766/SRR13334766_2.fastq.gz

ftp.sra.ebi.ac.uk/vol1/fastq/SRR133/069/SRR13334769/SRR13334769_2.fastq.gz

ftp.sra.ebi.ac.uk/vol1/fastq/SRR133/070/SRR13334770/SRR13334770_2.fastq.gz

ftp.sra.ebi.ac.uk/vol1/fastq/SRR133/073/SRR13334773/SRR13334773_2.fastq.gz

ftp.sra.ebi.ac.uk/vol1/fastq/SRR133/074/SRR13334774/SRR13334774_2.fastq.gz

ftp.sra.ebi.ac.uk/vol1/fastq/SRR133/075/SRR13334775/SRR13334775_2.fastq.gz

ftp.sra.ebi.ac.uk/vol1/fastq/SRR133/078/SRR13334778/SRR13334778_2.fastq.gz

ftp.sra.ebi.ac.uk/vol1/fastq/SRR133/082/SRR13334782/SRR13334782_2.fastq.gz

ftp.sra.ebi.ac.uk/vol1/fastq/SRR133/083/SRR13334783/SRR13334783_2.fastq.gz

ftp.sra.ebi.ac.uk/vol1/fastq/SRR133/084/SRR13334784/SRR13334784_2.fastq.gz

ftp.sra.ebi.ac.uk/vol1/fastq/SRR133/086/SRR13334786/SRR13334786_2.fastq.gz

ftp.sra.ebi.ac.uk/vol1/fastq/SRR133/087/SRR13334787/SRR13334787_2.fastq.gz

ftp.sra.ebi.ac.uk/vol1/fastq/SRR133/068/SRR13334768/SRR13334768_2.fastq.gz

ftp.sra.ebi.ac.uk/vol1/fastq/SRR133/072/SRR13334772/SRR13334772_2.fastq.gz

ftp.sra.ebi.ac.uk/vol1/fastq/SRR133/077/SRR13334777/SRR13334777_2.fastq.gz

ftp.sra.ebi.ac.uk/vol1/fastq/SRR133/080/SRR13334780/SRR13334780_2.fastq.gz

ftp.sra.ebi.ac.uk/vol1/fastq/SRR133/081/SRR13334781/SRR13334781_2.fastq.gz

ftp.sra.ebi.ac.uk/vol1/fastq/SRR133/085/SRR13334785/SRR13334785_2.fastq.gz

ftp.sra.ebi.ac.uk/vol1/fastq/SRR133/089/SRR13334789/SRR13334789_2.fastq.gz

ftp.sra.ebi.ac.uk/vol1/fastq/SRR133/065/SRR13334765/SRR13334765_2.fastq.gz

ftp.sra.ebi.ac.uk/vol1/fastq/SRR133/067/SRR13334767/SRR13334767_2.fastq.gz

ftp.sra.ebi.ac.uk/vol1/fastq/SRR133/071/SRR13334771/SRR13334771_2.fastq.gz

ftp.sra.ebi.ac.uk/vol1/fastq/SRR133/076/SRR13334776/SRR13334776_2.fastq.gz

ftp.sra.ebi.ac.uk/vol1/fastq/SRR133/079/SRR13334779/SRR13334779_2.fastq.gz

**HISAT2 ALIGNMENT STATISTICS FOR *F. hepatica* DATASETS PRJEB9655 & PRJEB6904**

952626590 reads; of these:

952626590 (100.00%) were paired; of these:

189652173 (20.27%) aligned concordantly 0 times

690040845 (72.17%) aligned concordantly exactly 1 time

72933572 (7.56%) aligned concordantly >1 times

----

189652173 pairs aligned concordantly 0 times; of these:

9182776 (5.01%) aligned discordantly 1 time

----

180469397 pairs aligned 0 times concordantly or discordantly; of these:

360938794 mates make up the pairs; of these:

268446466 (73.06%) aligned 0 times

78967888 (23.05%) aligned exactly 1 time

13524440 (3.89%) aligned >1 times

85.53% overall alignment rate
