# Supplementary material for: Discovery of long non-coding RNAs in the liver fluke, Fasciola hepatica
Source: PLoS Negl Trop Dis. 2023 Sep 28;17(9):e0011663. doi: 10.1371/journal.pntd.0011663 (PMC10564125; doi:10.1371/journal.pntd.0011663)
Supplement: S11 Dataset — Each lncRNA was measured across three life-stage libraries: Adult Fasciola hepatica, 21 day in vitro juvenile F. hepatica (ivt) and 21 day in vivo juvenile F. hepatica (ivv). Each datapoint represents the mean±SEM of at least three biological replicates. Under each graph, the primer sets used for ddPCR amplification are indicated. (PDF) [file pntd.0011663.s011.pdf]

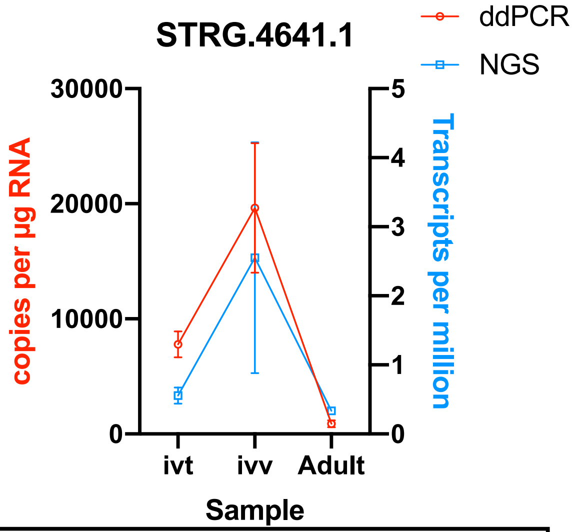

STRG.4641_F: CCGTTAGAGTTTGCTTCCCTG
STRG.4641_R: ATTGGGGTGACAGTAGTGAT

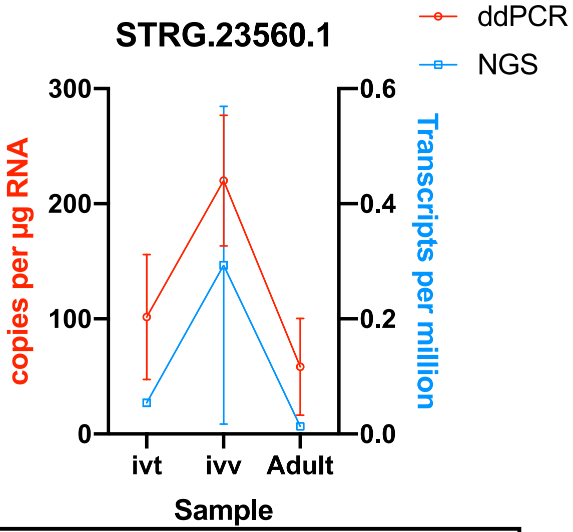

STRG.23560_F: ATTCCGTGTCTACGGTTTCG
STRG.23560_R: ACCAATGTGTGTTTGGTGCT

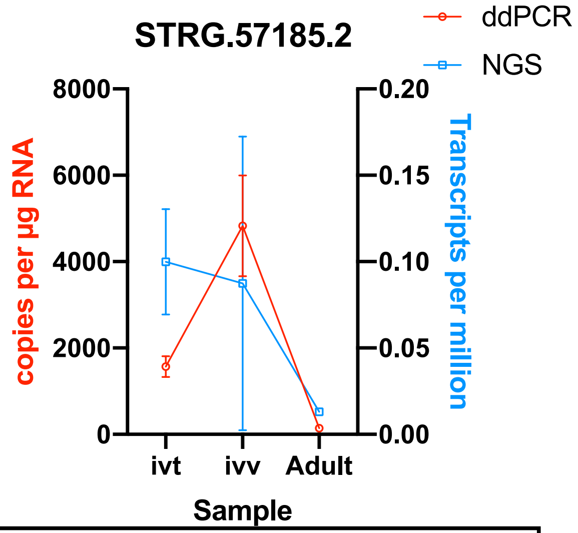

STRG.51785_F: TATCGACGTGTGGTGTGTAG
STRG.51785_R: CGGTCCAGATGTGTAGTCCA

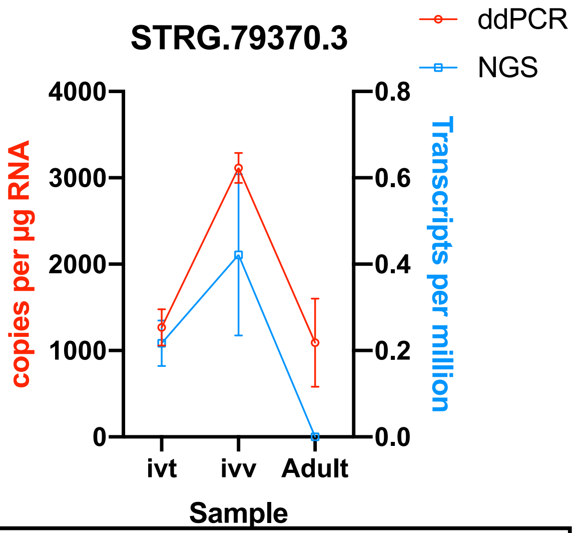

STRG.79370_F: TGACGCGTCTACTTACAGAACG
STRG.79370_R: TTGACACAGATGCCGCTTT

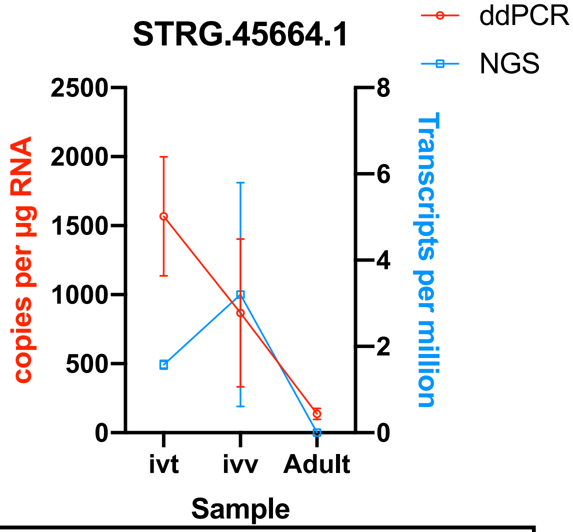

STRG.45664_F: GGCAAATTCGAAGGTCAAAG
STRG.45664_R: GCACTGATGCCATCTGACTC

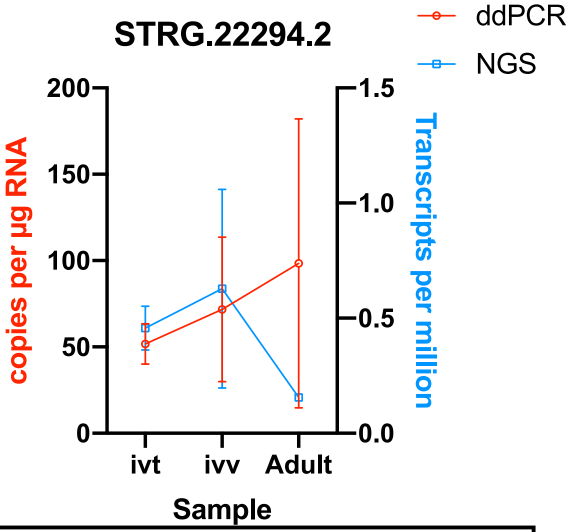

STRG.22294_F: TGTACTCGGGAAAATCGATGC
STRG.22294_R: TCCACTTCTCGAGGACCCTC
